# *Mycoplasma pneumoniae* Infection Associated C3 Glomerulopathy Presenting as Severe Crescentic Glomerulonephritis

**DOI:** 10.1155/2021/6295543

**Published:** 2021-09-27

**Authors:** Lalani De Silva, Dinesha Jayasinghe, Priyani Amarathunga

**Affiliations:** Department of Pathology, Faculty of Medicine, University of Colombo, Colombo, Sri Lanka

## Abstract

C3 glomerulopathy (C3GP) is a group of diseases caused by a deregulated complement system, which encompasses both dense deposit disease and C3 glomerulonephritis. Renal manifestations of C3GP are primarily of proliferative glomerulonephritis, and only a few case reports of crescentic glomerulonephritis (CGN) in association with C3GP are available. Here is a case of an adult South-Asian female, who was diagnosed as seropositive acute *Mycoplasma pneumoniae* infection, with associated systemic manifestations, including immune-type extravascular haemolysis and nephrotic range proteinuria. Subsequent renal biopsy revealed CGN with disrupted Bowman's capsules and necrotizing lesions. Immunofluorescence showed coarse granular mesangial C3 deposits with negative IgM, IgG, IgA, and C1q. The immunomorphological phenotype raised two possibilities including C3GP and infection-related glomerulonephritis (IRGN). Persistent proteinuria with no evidence of resolution even after 6 months of follow-up favoured C3GP over IRGN. The patient proceeded to end-stage renal failure requiring renal replacement despite aggressive immunosuppression. This case illustrates the rare association of CGN with C3GP induced by *Mycoplasma pneumoniae* infection, highlighting the importance of correct diagnosis as well as timely identification of triggering factors in CGN on patient outcome.

## 1. Introduction

Complement deposits are seen in virtually all types of glomerulonephritis [1]. Most of the complement-mediated renal diseases are caused as a secondary event following immune complex-mediated injury, such as membranoproliferative glomerulonephritis, lupus nephritis, antiphospholipid syndrome, IgA nephropathy, and cryoglobulinaemic glomerulonephritis. Primary complement-mediated injury is seen in C3 glomerulopathy (C3GP) and some infection-related glomerulonephritis (IRGN) including atypical haemolytic uremic syndrome (HUS) [1, 2]. C1q disease, although diagnosed based on dominant C1q deposits in immunofluorescence, is considered as a disease mediated by immune complexes rather than a disease of complement deregulation; thus, immune complexes are invariably identified in immunofluorescence studies [1–3]. CGN with isolated C3 deposits in immunofluorescence is a rare type of pauci-immune CGN. This incorporates most of the C3 glomerulopathy cases and occasional cases of resolving IRGN [4].

Herein is a case with severe crescentic glomerulonephritis showing isolated C3 deposits in immunofluorescence induced by *Mycoplasma pneumoniae* lung infection.

## 2. Case Presentation

A 58-year-old lady presented to a medical unit in a tertiary-care hospital with a four-day history of fever with chills and cough, associated with vomiting and loss of appetite. She denied any symptoms related to renal dysfunction prior to this admission. Basic investigations (full blood count, urine full report, ECG, serum creatinine, and AST/ALT) done a few years back for a medical checkup were within normal limits.

On examination, she was pale and had mild icterus and bilateral ankle oedema. Her hemodynamic parameters were normal (pulse rate: 86 beats/min, regular, good volume; blood pressure: 130/90 mmHg). There was mild splenomegaly with no hepatomegaly or free fluid. Chest examination revealed bilateral basal end-inspiratory crackles.

Initial investigations showed anaemia (hemoglobin: 7.5 g/dl), neutrophil leukocytosis (total leukocyte count: 25000/mm^3^; neutrophils: 90%), elevated C-reactive protein (28 U/l) and ESR (170 mm/1st hour), indirect hyperbilirubinaemia (total bilirubin: 22.3 mg/l; direct fraction: 5.8 mg/l), and high serum creatinine (174 mmol/l). Urine examination revealed nephrotic range proteinuria and microscopic haematuria. Chest X-ray showed diffuse, reticular, ground-glass opacity in the bilateral lung bases, more in the left side, suggestive of interstitial pneumonia. She was started on a second-generation cephalosporin, in addition to basic supportive care and antipyretic therapy.

Based on the findings of pallor, icterus, splenomegaly, anaemia, and indirect hyperbilirubinaemia, an extravascular haemolytic anaemia was suspected. A subsequent blood picture revealed normochromic normocytic red blood cells with cold-type autoagglutination, neutrophil leukocytosis with toxic change, and normal platelets. Positive Coombs test turned the diagnosis towards immune-type haemolytic anaemia. Prednisolone 40 mg daily was started on the 6^th^ day of admission as a treatment for immune haemolytic anaemia.

Despite antibiotic treatment, general supportive care, and transfusion of three pints of red cell concentrate, the patient continued to deteriorate with progressive leukocytosis (total leukocyte count: 25000/mm^3^ to 48000/mm^3^), anaemia (hemoglobin: 7.5 g/dl to 6.2 g/dl), and rising serum creatinine (174 mmol/l to 214 mmol/l) over 10 days. Additional laboratory workup revealed progressive nephrotic range proteinuria (561 mg/dl, urine protein/creatinine ratio: 837 mg/mmol) and positive mycoplasma IgM antibody titer (1/320). Antinuclear antibody, DsDNA, antistreptolysin O titer (ASOT), hepatitis B and C, VDRL, and HIV serology were negative. Both antimyeloperoxidase ANCA and antiproteinase-3 ANCA were negative. Bacteriological cultures of urine and blood were sterile. 2D-echocardiogram was performed to exclude the possibility of infective endocarditis and was compatible with mild myocarditis and global ventricular dysfunction with no evidence of infective endocarditis. A macrolide was added in view of positive mycoplasma serology, and her respiratory symptoms were markedly improved in a few days.

**Figure 1:**
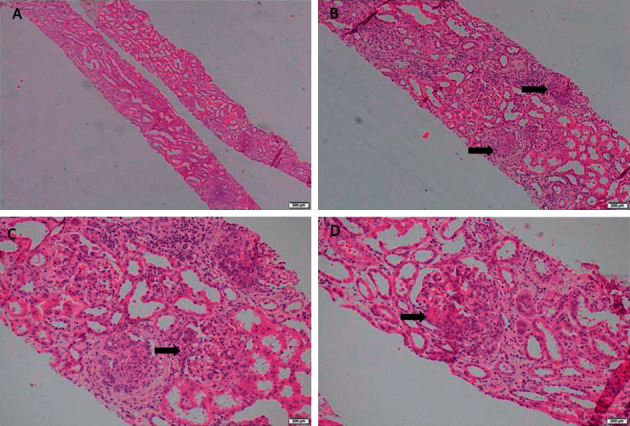
Microscopic images of the renal biopsy (H&E). (a) The biopsy was composed of two cores of renal tissue (40x). (b) It showed diffuse endocapillary proliferative glomerulonephritis with epithelial crescents (arrow) (100x). (c) Some glomeruli showed neutrophil infiltration (arrow) (400x) and (d) necrotizing lesions (arrow) (400x).

**Figure 2:**
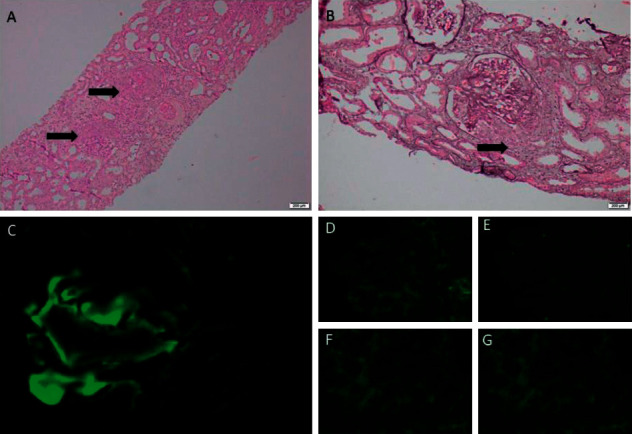
Periodic acid-Schiff (a) and silver (b) stained sections showed disruptive crescents extending beyond the limit of Bowman's capsule (arrows) (400x). (c) Immunofluorescence of C3 showed coarse granular mesangial deposits (400x). Immunofluorescence was negative for IgG (d), IgA (e), IgM (f), and C1q (g).

Because of persistent nephrotic range proteinuria and rising serum creatinine, a percutaneous renal biopsy was performed on the 12^th^ day of hospital admission. The biopsy showed diffuse endocapillary proliferative glomerulonephritis with crescents. These crescents expanded beyond the limit of Bowman's capsule disrupting the glomerular outline (Figure 1). There were focal leukocyte infiltration, karyorrhexis, and necrotizing lesions. No evidence of capillary microthrombi or vasculitis. A few tubules showed red cell casts. Immunofluorescence examination revealed isolated C3 deposits in the mesangium with negative IgA, IgG, IgM, and C1q. It was concluded as ANCA-negative pauci-immune CGN with isolated C3 deposits (Figure 2).

As the patient did not respond to oral prednisolone, she was started on intravenous methylprednisolone with which her serum creatinine level became normal. However, she continued to have nephrotic range proteinuria and the clinical decision was to start her on cyclophosphamide pulse therapy. During the follow-up, she continued to have proteinuria even with cyclophosphamide therapy for more than 6 months. As the possibility of IRGN is unlikely to be continued more than 8-week duration, the clinical diagnosis of C3GP was made. However, the unavailability of specific genetic testing to identify the defect in the complement cascade precluded further narrowing down of possibilities in arriving at a specific definitive diagnosis.

## 3. Discussion

C3 glomerulopathy was identified as a specific pathological entity in 2012 by a group of experts emphasizing that it is a heterogeneous group of diseases with isolated/dominant C3 deposits. It requires further evaluation to identify the specific complement defects in the cascade, if any, to approach a specific anticomplement targeted therapy; meanwhile, these targeted complement inhibitors are being developed [4].

The term C3GP incorporates two specific entities including dense deposit disease (DDD) and C3 glomerulonephritis. Distinction of these two is solely based on the morphology of the deposits on electron microscopy (EM) as the clinical and light microscopic features are almost identical. The light microscopy of these two entities ranges from mesangioproliferative, membranoproliferative, and endocapillary proliferative to severe CGN [4, 5]. The typical EM findings of DDD are the presence of highly electron-dense, osmophilic deposits within glomerular basement membranes forming band-like or “sausage-like” shapes with punctated skipped areas of more normal looking glomerular basement membranes. When the EM appearance of C3 deposits fails to achieve those specific features, by means of being less electron-dense, ill-defined, more confluent, or more rounded, the disease is classified as C3 glomerulonephritis [4–6].

Differentiation of these two entities in CGN is prognostically important [7]. As observed in one study, patients with C3 glomerulonephritis presented with severe necrotizing and crescentic glomerulonephritis showed more stable renal function following immunosuppressive therapy, compared with more patients with DDD presented with crescents progressed to end-stage renal failure requiring renal transplant, despite various immunosuppressive modalities. Two of them had recurrence of DDD in the grafted kidney [8].

The pathogenic mechanism of C3GP is the uncontrolled activation of the complement cascade through an alternative pathway, which can be either inherited/familial or acquired [9]. Genetic alterations leading to familial forms of C3GP include mutations in complement regulatory proteins such as factor H, factor I, and CD46, gain-of-function mutation of complement activating proteins such as factor B and C3, and genomic rearrangement within complement factor H related genes which includes five separate genes, namely, CFHR1, CFHR2, CFHR3, CFHR4, and CFHR5 [9]. The acquired form is resulted from uncontrolled activation of complement mostly through autoimmune mechanisms. C3 nephritic factor, an autoantibody stabilizing alternative pathway C3 convertase C3bBb, anti-factor B autoantibody, and anti-factor H autoantibody are some of the identified autoantibodies [9]. In addition, monoclonal gammopathy also plays a role in the acquired form of C3GP [4, 9].

Genetic and serological testing to identify defects in the complement cascade requires highly specialized laboratory facilities and expert interpretation which are not available in most of the developing countries. Therefore, in most of the instances, evaluation of patients suspecting C3GP is halted at this level due to a lack of resources [4].

Current approach to specific treatment for C3GP is controversial due to the lack of adequate data on therapeutic trials. Steroid therapy, although a common practice, fails to show an effective response especially in DDD. Anti-C5 therapy (e.g., eculizumab) has shown some beneficial effects. Future therapeutic approach is heading towards the development of targeted therapy for specific complement defects, thereby achieving a complete remission of the disease [10, 11].

Clinical presentation of this patient raised the possibility of IRGN. Although the usual presentation of IRGN is an acute nephritic syndrome, approximately a third of patients can be presented with nephrotic range proteinuria [12]. In addition, the associated extrarenal manifestation and serum hypocomplementaemia also favoured IRGN. The possibility of IRGN due to mycoplasma infection is raised based on clinical symptoms of pulmonary (interstitial pneumonia) and extrapulmonary (myocarditis and extravascular haemolysis) manifestations and positive mycoplasma IgM antibodies together with supportive serological evidence (neutrophil leukocytosis, high CRP/ESR, positive direct Coombs test, cold-type autoimmune haemolytic anaemia, and hypocomplementaemia).

Renal manifestations of mycoplasma infection include a spectrum of tubulointerstitial nephritis and glomerulonephritis, including mesangioproliferative glomerulonephritis and endocapillary proliferative glomerulonephritis [13, 14]. Although rare, a few cases of CGN are also reported [15, 16]. CGN with isolated C3 deposition as seen in this patient is extremely rare, with only a single case reported in the literature [17]. As reported by Mao et al., an 8-year-old girl presented with a one-week history of oedema, haematuria, and anaemia with positive serology for both mycoplasma and ASOT. The renal biopsy showed CGN with isolated C3 deposition and subepithelial dense material deposition on EM. She was successfully treated with immunosuppressive therapy with methylprednisolone and cyclophosphamide and showed an excellent clinical outcome [17]. However, the possibility of IRGN due to streptococcus infection should also be considered in this scenario.

Resolving stage of IRGN can show isolated C3 deposits in immunofluorescence similar to the findings of C3GP. Therefore, the differentiation of these two is solely based on clinical follow-up. Patients with IRGN are expected to be completely recovered from renal derangement in 8–12 weeks, while patients with C3GP show persistent proteinuria even with immunosuppressive therapy [4]. Considering the fact that this patient is having persistent heavy proteinuria over 6-month duration, even with advanced immunosuppressive therapy, C3GP is considered the most possible diagnosis over IRGN.

Extravascular haemolysis associated with acute kidney injury can be seen in atypical HUS. Atypical HUS contributes approximately 10% of all HUS and includes cases which are not related to Shiga toxins of enteric bacteria [18]. Both C3GP and atypical HUS are related to defects in the alternative pathway of complement cascade and show similar pathogenesis. Over 40% of atypical HUS patients show genetic mutations in the genes of complement proteins and of its regulatory proteins or autoantibodies to factor H, which makes it is practically impossible to differentiate atypical HUS from C3GP even with extensive genetic analysis [18, 19]. However, atypical HUS is unlikely in this patient because of the positive Coombs test (atypical HUS is a nonimmune haemolytic disease and in turn Coombs negative), absence of thrombocytopaenia (a key presenting feature), and absence of capillary microthrombi in the renal biopsy.

Considering overall clinical and immunomorphological features, C3GP was considered as the most possible diagnosis in this patient. We suggest that mycoplasma infection would have triggered the activation of immunosusceptibility of C3 complement pathway in this patient. However, limited resources precluded the detection of specific defects in complement cascade.

In conclusion, CGN is a disease process rather than a specific disease, due to a variety of causative agents. Identification of specific aetiology early in the process is important therapeutically to make clinical decisions on treatment modality and thereby to achieve a better outcome. Although biopsy diagnosis with basic light microscopic categorization of CGN is fundamental, supplementary evidence by immunofluorescence and EM is invaluable in arriving at a specific diagnosis. Knowledge on the genetic basis of the disease and its practical application for diagnostic and therapeutic process is important nowadays in evaluating patients with CGN, although the required facilities are not well established in most places.

## Data Availability

Data pertaining to this case report can be obtained from the corresponding author upon a reasonable request.
